# Supplementary material for: Cryopreservation of Cyanobacteria and Eukaryotic Microalgae Using Exopolysaccharide Extracted from a Glacier Bacterium
Source: Microorganisms. 2021 Feb 15;9(2):395. doi: 10.3390/microorganisms9020395 (PMC7918967; doi:10.3390/microorganisms9020395)
Supplement: Supplementary file 1 [file microorganisms-09-00395-s001.pdf]

Supplemental figures

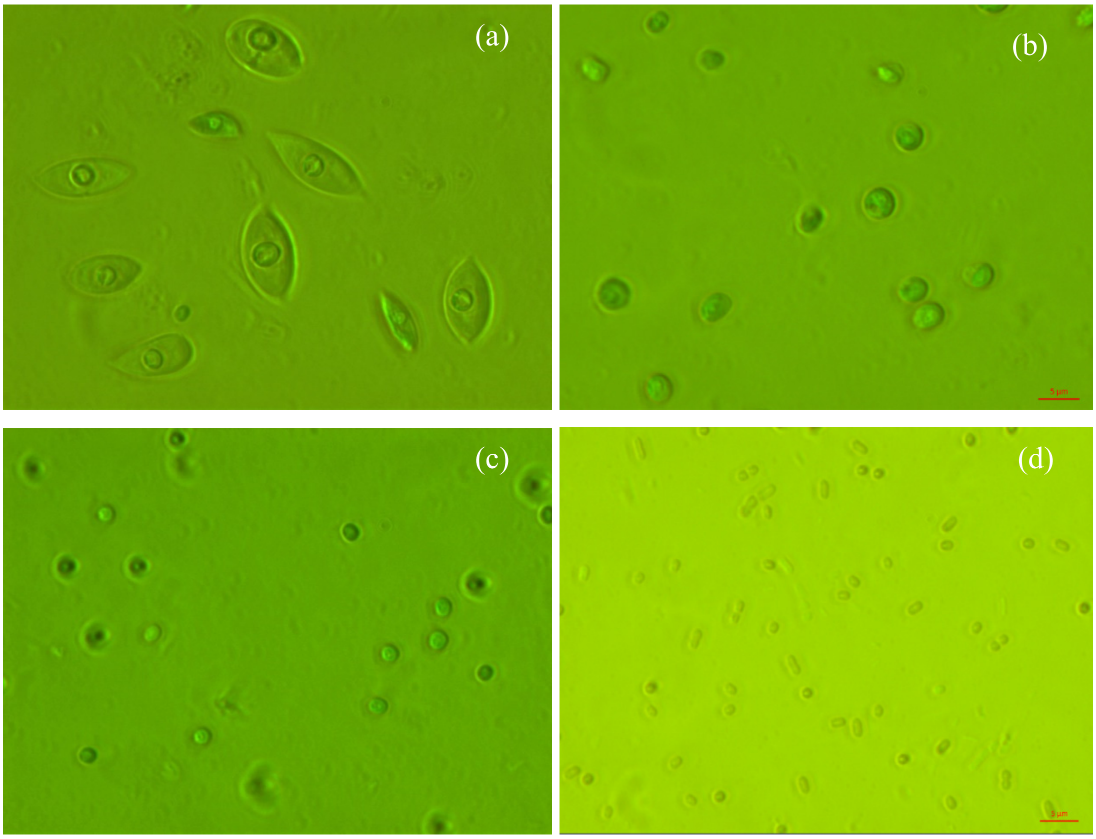

Figure S1. Cell morphology of the microalgal and cyanobacterial strains under 100X light microscopy (a) *Scenedesmus obliquus* HTB1 (b) *Chlorella vulgaris* UTEX 2714 (c) *Microcystis aeruginosa* PCC 7806 (d) *Synechococcus* sp. CBW1003 (cell morphology of CB0101 is similar to CBW1003, image not shown).

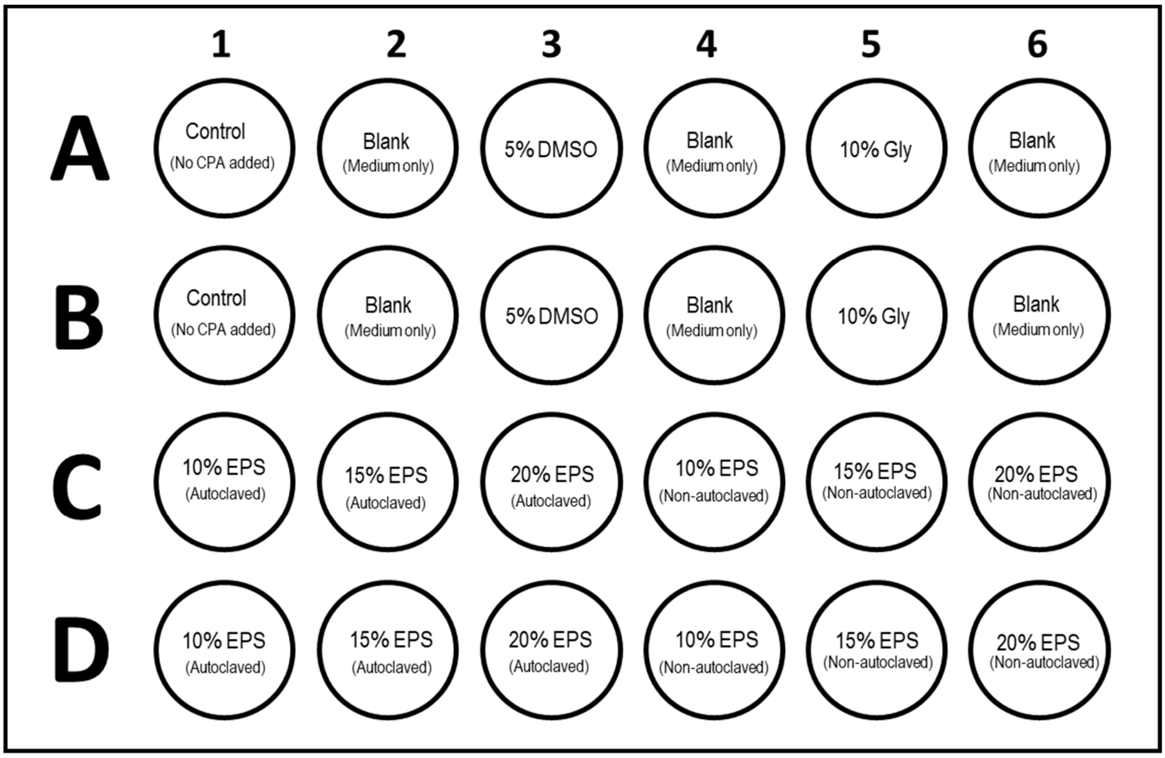

Figure S2. Picture of a 24 well plate used for biomass recovery test of the microalgal and cyanobacterial strains. Well A1/B1 represents control (cultures preserved with no CPA), A3/B3 5% DMSO, A5/B5 10% glycerol, A2/B2 and A5/B5 culture medium only (blank), C1/D1 10% autoclaved EPS, C2/D2 15% autoclaved EPS, C3/D3 20% autoclaved EPS, C4/D4 10% non-autoclaved EPS, C5/D5 15% non-autoclaved EPS, C6/D6 20% non-autoclaved EPS.

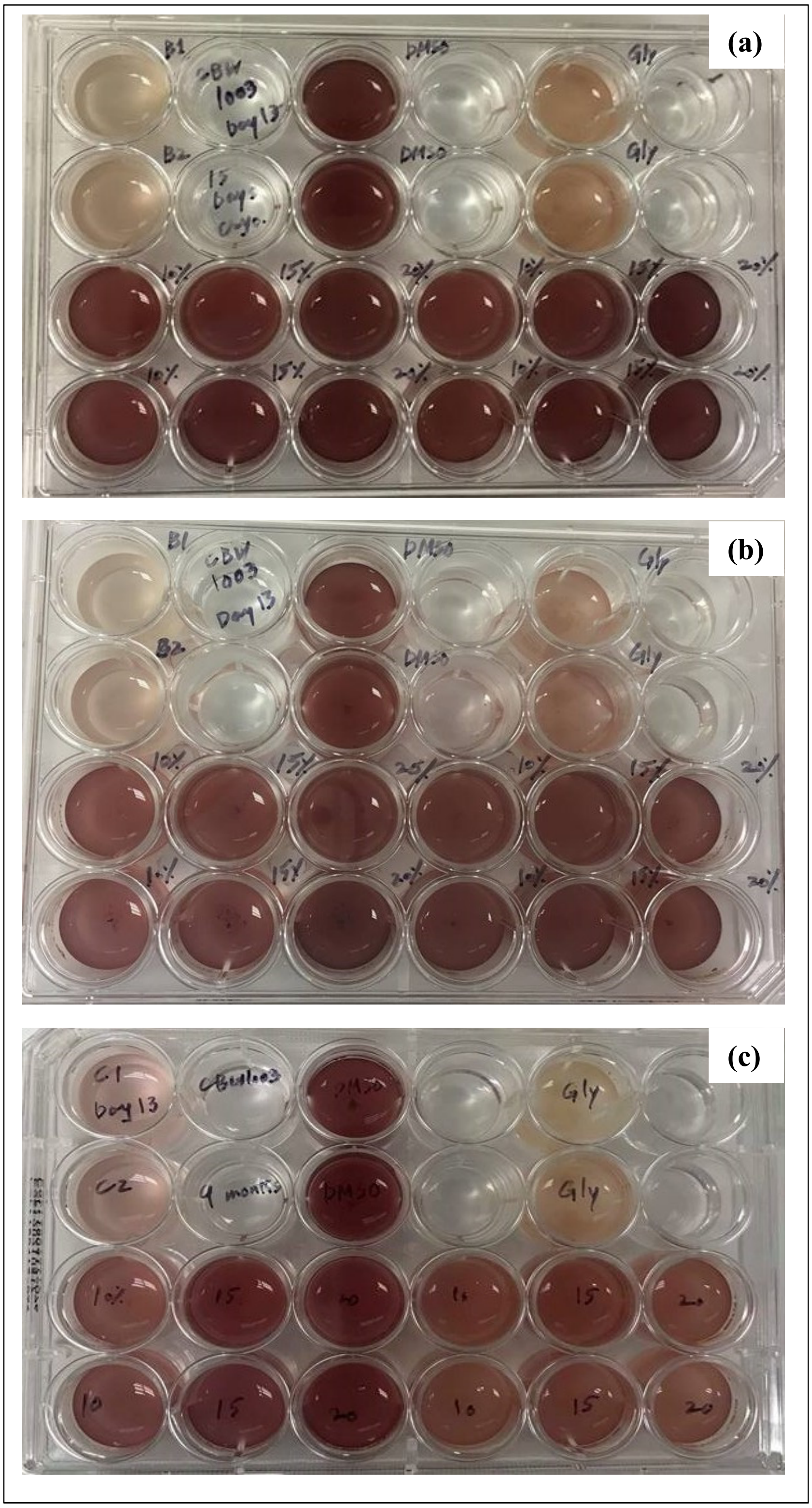

Figure S3. Growth recovery for *Synechococcus* sp. CBW1003 after cryopreservation (a) 5 days, (b) 15 days, and (c) 9 months, in the 24 well plates. The layout of treatments and controls on these plates refers to Fig. S1. This figure corresponds to Fig. 1.

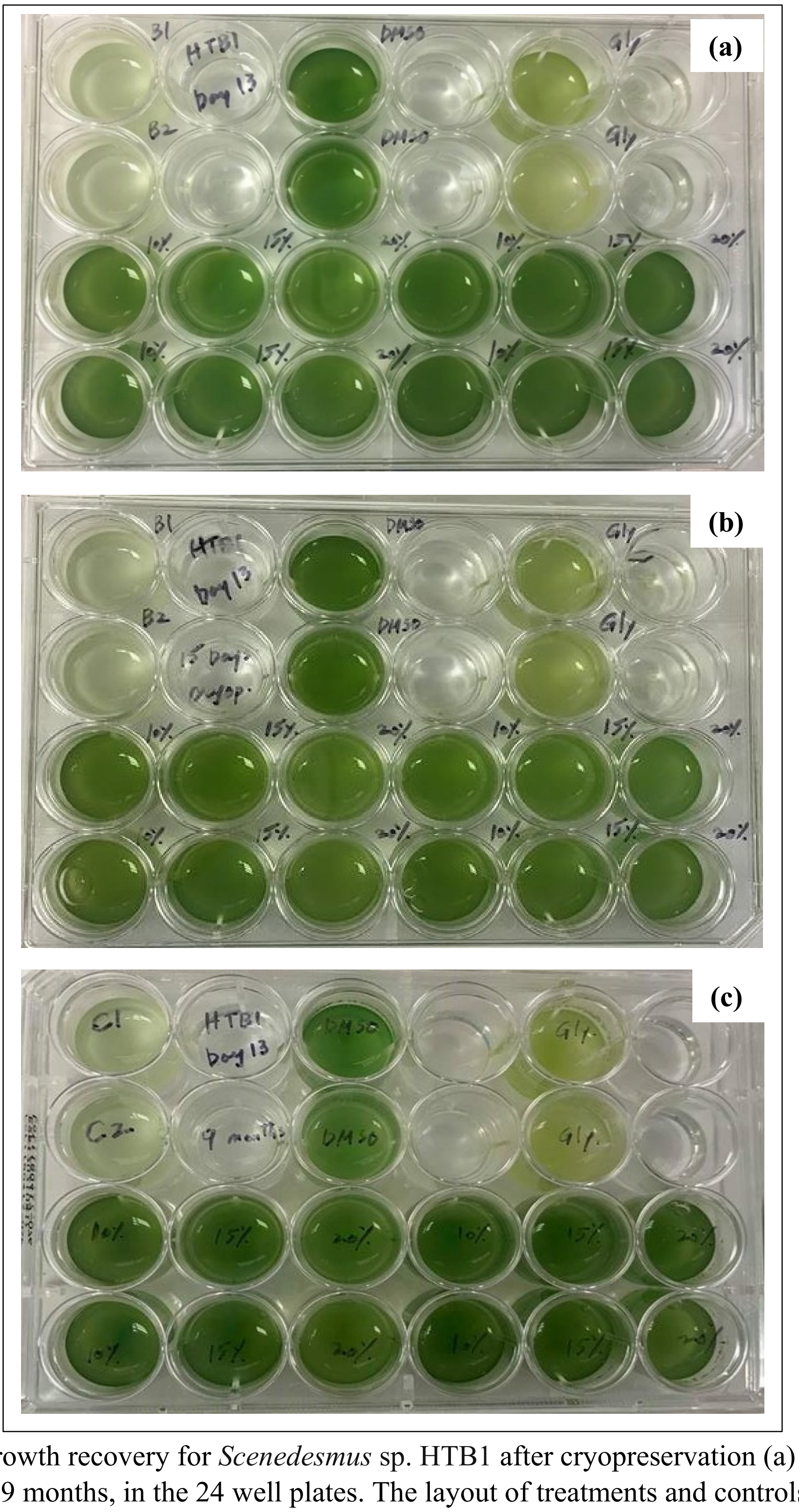

Figure S4. Growth recovery for *Scenedesmus* sp. HTB1 after cryopreservation (a) 5 days, (b) 15 days, and (c) 9 months, in the 24 well plates. The layout of treatments and controls on these plates refers to Fig. S1.This figure corresponds to Fig. 2.

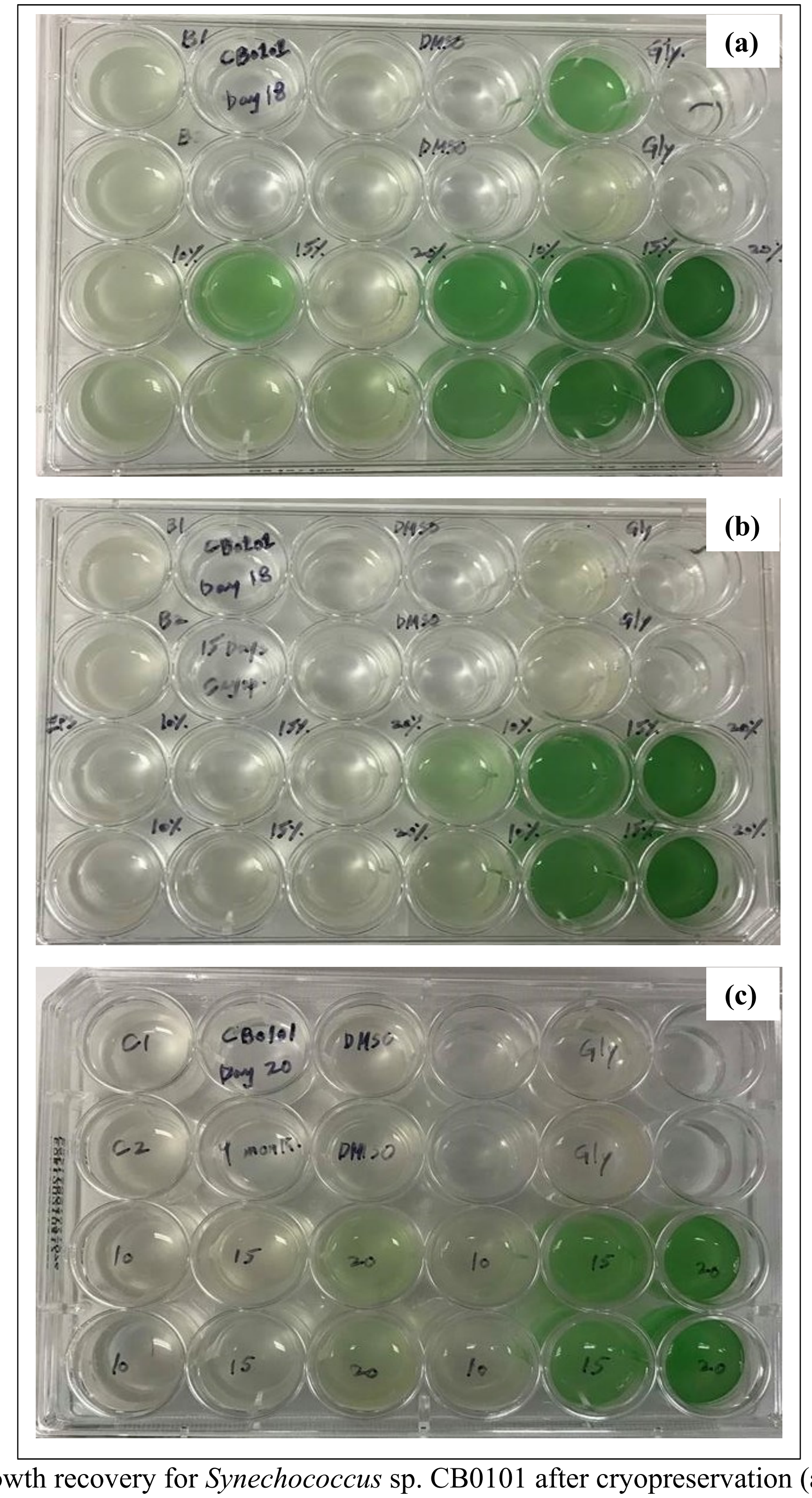

Figure S5. Growth recovery for *Synechococcus* sp. CB0101 after cryopreservation (a) 5 days, (b) 15 days, and (c) 9 months, in the 24 well plates. The layout of treatments and controls on these plates refers to Fig. S1. This figure corresponds to Fig. 3.

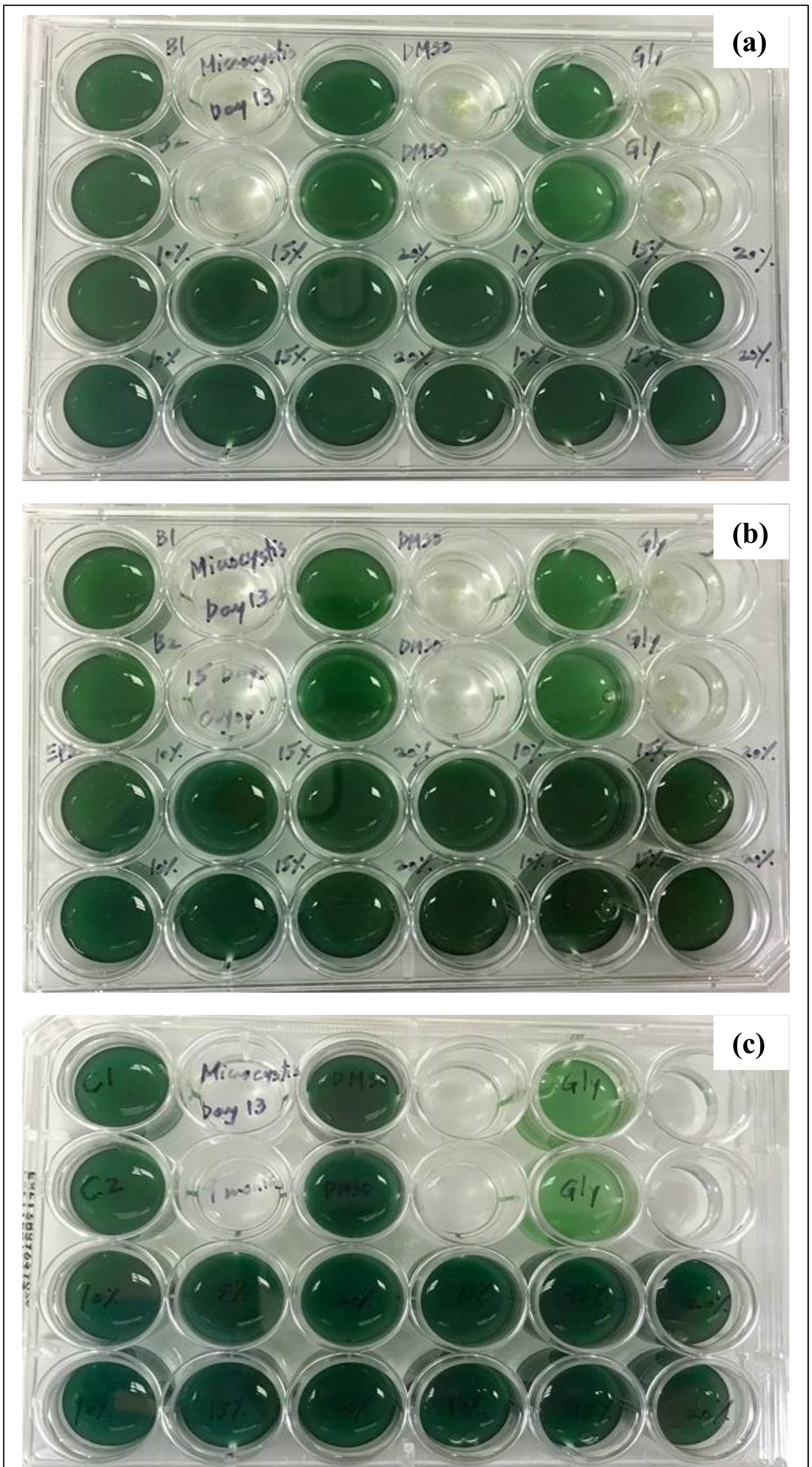

Figure S6. Growth recovery for *Microcystis aeruginosa* PCC 7806 after cryopreservation (a) 5 days, (b) 15 days, and (c) 9 months, in the 24 well plates. The layout of treatments and controls on these plates refers to Fig. S1. This figure corresponds to Fig. 4.

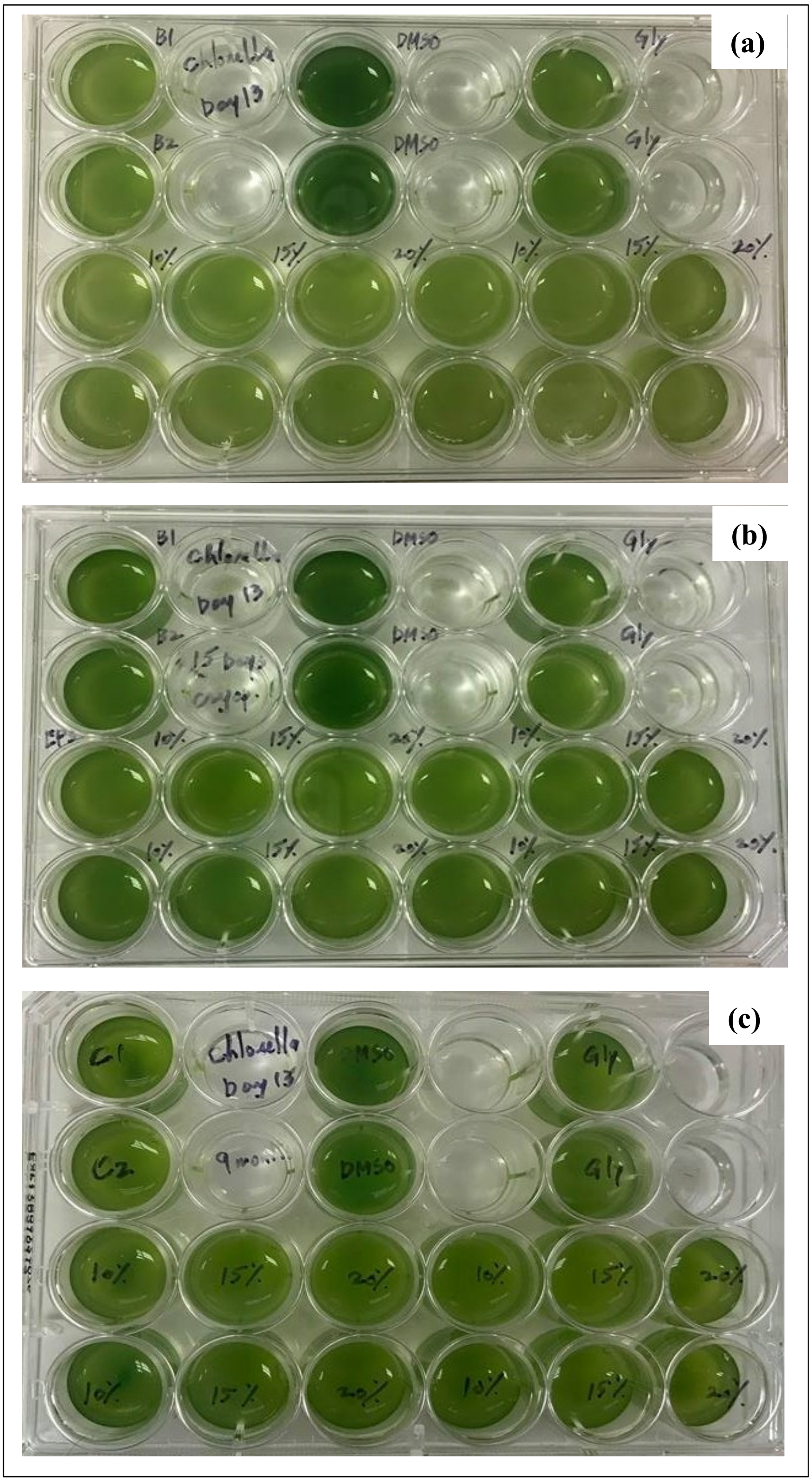

Figure S7. Growth recovery for *Chlorella vulgaris* after cryopreservation (a) 5 days, (b) 15 days, and (c) 9 months, in the 24 well plates. The layout of treatments and controls on these plates refers to Fig. S1. This figure corresponds to Fig. 5.
